# Guidelines for authors and reviewers on antibody use in physiology studies

**DOI:** 10.1152/ajpheart.00512.2017

**Published:** 2018-01-05

**Authors:** Heddwen L. Brooks, Merry L. Lindsey

**Affiliations:** ^1^Department of Physiology, Pharmacology and Medicine, Sarver Heart Center, College of Medicine, University of Arizona, Tucson, Arizona; ^2^Mississippi Center for Heart Research, Department of Physiology and Biophysics, University of Mississippi Medical Center, Jackson, Mississippi; ^3^Research Service, G.V. (Sonny) Montgomery Veterans Affairs Medical Center, Jackson, Mississippi

**Keywords:** antibody, immunoblot analysis, immunoblotting, immunohistochemistry, rigor and reproducibility

## Abstract

Antibody use is a critical component of cardiovascular physiology research, and antibodies are used to monitor protein abundance (immunoblot analysis) and protein expression and localization (in tissue by immunohistochemistry and in cells by immunocytochemistry). With ongoing discussions on how to improve reproducibility and rigor, the goal of this review is to provide best practice guidelines regarding how to optimize antibody use for increased rigor and reproducibility in both immunoblot analysis and immunohistochemistry approaches.

Listen to this article’s corresponding podcast at http://ajpheart.podbean.com/e/guidelines-on-antibody-use-in-physiology-studies/.

## INTRODUCTION

Measuring protein quality and quantity is an important component in cardiovascular physiology studies, as a means to provide mechanistic insights into the cellular and molecular signaling pathways involved. For immunoblot analysis, antibodies can tell us whether the protein present in a sample is full length or fragmented, whether it is in a pro form or processed, or whether it has undergone posttranslational modifications such as phosphorylation. Thus, the quality of the protein of interest can be assessed by comparing the expected molecular weight of a protein with the actual band size on the blot. The abundance or quantity of a protein of interest can be measured and compared between samples. For immunohistochemistry, quality can be assessed by comparing the expected protein location and expression level with the actual expression pattern. This tells us whether the protein is intracellular or extracellular, whether it is membrane bound or nuclear localized, or whether trafficking has occurred. With increasing concerns over reproducibility and rigor, best practice guidelines are needed regarding the use of antibodies to monitor protein in experimental conditions. Specifically, the information included in a manuscript is vital to provide reviewers and readers with sufficient details to assess data quality and allow the next scientist to compare their future results with those in the literature.

Antibody misuse is a barrier to improved rigor and reproducibility. It is estimated that up to 50% of studies are not reproducible as published; of these, ~35% of the issue can be attributed to biological reagents, including antibody use (1, 5, 12, 33, 46). Antibody validation is not just the responsibility of the source; investigators need to provide application-specific validation. Antibody validation includes documenting sensitivity (what dilution or concentration of the antibody is needed to recognize the antigen), specificity (evaluate whether the antibody recognizes anything else in the sample), and reproducibility (antibody gives consistent results across different blotting methods or fixation protocols). For example, with immunoblot analysis it is important to give details on the percentage of gel used, sample preparation, and transfer methods, in addition to antibody details.

Because most antibodies likely do not completely fulfill all of the above validation criteria, the focus of this guidelines article will be to offer guidance for both authors and reviewers on how to provide consistent results and review expectations. We provide both the minimum requirements that are highly recommended as well as extended requirements that provide irrefutable rigor and reproducibility. We focus mostly on antibodies that are commercially developed, as our analysis of *American Journal of Physiology-Heart and Circulatory Physiology* articles published from January to June 2017 demonstrated that these are more commonly used; however, we discuss parameters for antibodies that are developed internally by an individual laboratory. A major issue with antibody use is the assumption that commercial antibodies are prevalidated by the company, an assumption that varies widely on a case-by-case basis (3a).

Below, the sections are divided by use and include discussion on using antibodies for immunoblot analysis or immunohistochemistry. The extensive reference list serves as a resource to investigators new to the field. This guidelines article is a good companion to other recent *American Journal of Physiology-Heart and Circulatory Physiology* guidelines, including “Guidelines for experimental models of myocardial ischemia and infarction” (35), “Guidelines for measuring cardiac physiology in mice” (37), and “Recording sympathetic nerve activity in conscious humans and other mammals: guidelines and the road to standardization” (17).

## ANTIBODY USE IN *AMERICAN JOURNAL OF PHYSIOLOGY-HEART AND CIRCULATORY PHYSIOLOGY* ARTICLES

The *American Journal of Physiology-Heart and Circulatory Physiology* provides instructions to authors (http://www.physiology.org/author-info.experimental-details-to-report), which are summarized here. Authors are requested to provide details on antibody use, with details focused on antibody use in immunoblot studies. Information requested includes antibody source (company name and catalog number). One representative full blot should be provided as supplemental data for reviewers, detailing the validation of each antibody used in the study to demonstrate protein specificity. This should be done for every antibody that has not been previously tested, especially antibodies developed within a laboratory. Lanes on the blot should be labeled to note nonspecific and specific bands and positive and negative controls. This is an important component, as this is where you show that the antibody works as expected. The journal requests that exposure time be provided; this is especially important if samples are run across several gels, to show that gel-to-gel variation is minimized. If the data already exist, the reference for the publication can be given; if this reference is not from your own laboratory, then the assumption you make is that the validation was performed to your standards of quality. Image manipulation is not allowed by the *American Journal of Physiology-Heart and Circulatory Physiology*, and authors are advised that best practice is to not alter the gel or blot in any way. If gels, blots, or fields are grouped or rearranged, then spaces or dividing lines must be inserted to indicate where changes have been made, and any rearrangements should be detailed in the figure legend. Additionally, any adjustment to contrast, color balance, or brightness must be applied to the entire figure, and such adjustment cannot obscure or eliminate background. To selectively adjust one or only a few lanes is data manipulation. A major benefit of publishing in the *American Journal of Physiology-Heart and Circulatory Physiology* is that careful postacceptance review of figures occurs to ensure that any issues are corrected before publication (59). As we will highlight later in this review, these guidelines are not always followed, and additional guidelines may improve reproducibility efforts.

### Assessment of 2017 Published American Journal of Physiology-Heart and Circulatory Physiology Articles for Compliance with Current Guidelines

We assessed articles published in the *American Journal of Physiology-Heart and Circulatory Physiology* to determine whether adequate information was provided. A total of 139 articles were published in the *American Journal of Physiology-Heart and Circulatory Physiology* from January 1, 2015, to June 30, 2017, that met the search criteria (search done on June 30, 2017, using “Am J Physiol Heart Circ Physiol” and one of the following terms: “antibody,” “immunoblotting,” “Western,” “immunohistochemistry,” or “immunofluorescence”). Of these, two were review articles, one was a letter to the editor, and one was an editorial, giving a final total of 135 articles using antibodies to measure protein levels. These articles accounted for 14.6% of the 923 articles published in the *American Journal of Physiology-Heart and Circulatory Physiology* during this time period. The search did not include the use of antibodies for flow cytometry, ELISAs, or blocking studies. From the search results, we evaluated all articles published in 2017, for a total of 28 articles (2, 8–11, 13, 15, 16, 19, 21, 22, 26–28, 30, 32, 34, 41, 42, 44, 45, 47, 48, 50–52, 54, 58). Of these, 79% (22 of 28 articles) used immunoblot analysis, and 39% (11 of 28 articles) used immunohistochemistry (5 articles of the 28 evaluated used both techniques, 18%).

**Fig. 1.**
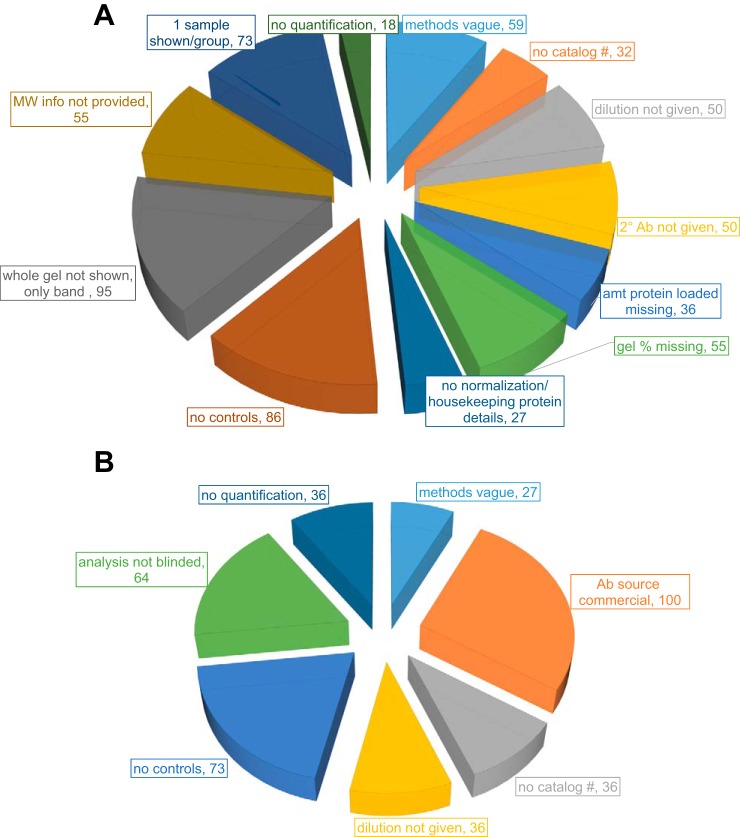
Literature assessment of antibody (Ab) use in articles published in the *American Journal of Physiology-Heart and Circulatory Physiology*. *A*: Ab use in immunoblot experiments. *B*: Ab use in immunohistochemistry experiments. amt, amount; MW, molecular weight.

Our results demonstrate that for immunoblot analysis, 95% of the articles showed images with only the band of interest and not any extra part of the blot, 86% were run with no controls, 73% showed only one representative sample per group, and 59% had vague methods details, which could make it difficult to reproduce the methodology in an independent study (see Fig. 1*A*, additional criteria evaluated). We are not criticizing these authors, as at least some of these comments apply to our own work. As a reference for immunoblotting methods, one publication had very well-described methods and could be used as a positive example (50).

For immunohistochemistry, our results demonstrated that 27% (3 of 11 articles) had vague methods, 73% (8 of 11 articles) were run with no controls, and 64% (7 of 11 articles) did not blind their analysis (see Fig. 1*B*, additional criteria evaluated). It is interesting that the instructions to authors given by the *American Journal of Physiology-Heart and Circulatory Physiology* are more detailed for immunoblot analysis, yet adequate details were more often provided for immunohistochemistry than for immunoblot analysis. We believe that this reflects concerted efforts by histology and pathology organizations, including the Histochemistry Society, the College of American Pathologists, and the American Society of Investigative Pathology, to establish clear guidelines with buy in from the scientific community (6, 7, 18). This indicates that authors are willing to follow instructions, provided that sufficient information is provided to allow them to do so. Given the low percentage of papers that blind the analysis for immunohistochemistry data, this may be an area for future instruction.

## IMMUNOBLOT ANALYSIS

Immunoblot analysis (also termed “Western blot analysis”) refers to the detection of a specific protein in a mixture of proteins obtained from tissue or cells. Protein extraction is performed to obtain a total protein pool from tissue or cell samples or the protein pool of interest is solubulized from serum, plasma, urine, or conditioned media. The protein sample is run on an SDS-polyacrylamide gel to separate proteins by molecular weight, transferred to a membrane, and probed by an antibody for the protein of interest. There are a number of resources to obtain immunoblotting protocols, and thus we will focus on antibody and presentation considerations (3, 24).

### Antibody Source, Dilutions, and Target Protein Concentrations

**Fig. 2.**
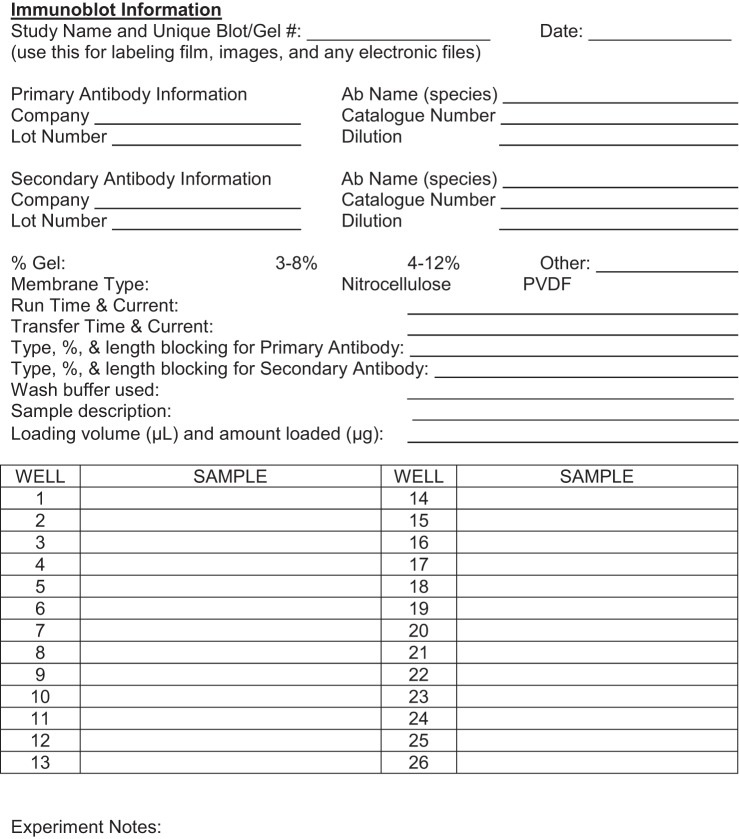
Sample laboratory notebook page for recording immunoblot experiments. Ab, antibody; PVDF, polyvinylidene difluoride.

Selecting which antibody to use is the most difficult part of the process, as there are a number of resources available for searching that can provide dozens of antibodies to select for any given antigen. Using websites that provide proof of validation is a good starting point, and Table 1 shows a number of resources with antibody information from hundreds of suppliers. Information on which antibody details should be recorded in your laboratory notebook and a template for recording antibody use are shown in Fig. 2. In our analysis of the 28 published papers in the *American Journal of Physiology-Heart and Circulatory Physiology*, it was common to see a lack of details on the antibody dilutions used and protein concentrations run on gels.

**Table 1.** Antibody resources

Site Name	Address	Information Provided
*Antibody search sites*		
Antibodypedia (4)	https://www.antibodypedia.com/	Portal for validated antibodies and antigens
The Antibody Registry	http://antibodyregistry.org/	Assigns unique and persistent identifiers to each antibody that can be referenced in publications
Antibody Resource	https://www.antibodyresource.com/	Provides a list of search sites, a catalog of catalogs
Antibody Review	http://www.antibodyreview.com/	Based on a Protein Knowledge Base called ProteinKB, a curated database containing 42,000 human, mouse, and rat proteins
Biocompare Antibody Search Tool	https://www.biocompare.com/Antibodies/	Has an excellent video that discusses reproducibility issues
CiteAb	https://www.citeab.com/	Largest antibody search engine, ranks antibodies by citation number
Linscott’s Directory of Immunological & Biological Reagents	https://www.linscottsdirectory.com/	Includes user reviews
*Society resources*		
AHo’s Amazing Atlas of Antibody Anatomy	https://www.bioc.uzh.ch/plueckthun/antibody/index.html	Has an “Antibodies for Beginners” section
American Physiological Society	“Guidelines for authors and reviewers on antibody use in physiology studies” (this article)	Details controls to be performed and information to be recorded to increase reproducibility of antibody use in physiology research
Anatomic Pathology Checklist	http://www.cap.org/apps/docs/laboratory_accreditation/checklists/anatomic_pathology_Sep07.pdf	Checklist used for inspection of laboratories by the Commission on Laboratory Accreditation of the College of American Pathologists
Antibody Society	http://www.antibodysociety.org/	International forum for field of recombinant antibodies; has on-site tools for antibody informatics
FASEB Enhancing Research Reproducibility	https://www.faseb.org/Portals/2/PDFs/opa/2016/FASEB_Enhancing%20Research%20Reproducibility.pdf	Section on research using antibodies provides recommendations

Note that sites for individual commercial resources are not listed here. FASEB, Federation of American Societies for Experimental Biology.

In the case of newly developed or noncommercial antibodies, additional information that should be provided includes the sequence used for the peptide or whether full-length recombinant protein was used, host, and bleed number. If a full-length recombinant protein was used, it is important to record the UniProt number as many different species and isoforms of the same protein exist. Also needed for a newly developed in-house antibody is blockade with the peptide to demonstrate specificity (29, 39). This is typically shown by running a dilution range of primary antibody concentrations (e.g., 1:500 to 1:10,000), secondary antibody concentrations (e.g., 1:500, 1:1,000, and 1:2,500), and target protein (e.g., 1, 5, and 25 μg) to demonstrate specificity. A complete list of controls that can be used for both immunoblot analysis and immunohistochemistry is shown in Table 2.

**Table 2.** Recommendations for controls

Control	Use	Type	Information Provided/Caveats	Priority
Known source tissue	IB/IHC	Positive	Antibody can recognize the antigen; easy and inexpensive control	High
Tissue or cells from null animal	IB/IHC	Negative	Evaluates nonspecific binding in the absence of the protein target	High
No primary antibody	IHC	Negative	Evaluates specificity of primary antibody binding to antigen; requires sufficient material; not needed for every sample	High
Reacting primary antibody with saturating amounts of antigen	IB/IHC	Negative	Absorption control to eliminate specific response; important control for untested antibody	Medium to low
Nonimmune serum from the same species as primary antibody	IB/IHC	Negative	Eliminates specific response	Low
No primary or secondary antibody	IHC	Negative	Evaluates label specificity for primary antibody; requires sufficient material; not needed for every sample	Low
Recombinant protein	IB	Positive	Antibody can recognize the antigen; high cost, especially for antibodies not routinely used	Low

IB, immunoblot analysis; IHC, immunohistochemistry.

### Primary Antibody Positive Controls Are Performed to Test Application-Specific Antibody Performance

Application-specific performance means that antibodies validated for use in histological examination may not recognize antigen in immunoblotting procedures, and vice versa. Positive controls include the recombinant protein and tissues or cells that are known to express the protein. Even if the company provides information regarding its antibody, a minimum positive control should be included.

Once (and if) an antibody has been validated to identify the correct protein with respect to specificity and sensitivity, the question then arises as to how to use it to evaluate a scientific question in cardiovascular disease. With respect to immunoblot analysis, this invariably involves the investigator using an immunoblot to demonstrate that the protein of specific interest increases or decreases following an experiment. This is the point where many of the *American Journal of Physiology-Heart and Circulatory Physiology* articles examined demonstrated a lack of reproducibility in presenting how abundance was quantified (Fig. 1*A*, quantification process and normalization).

### Protein Quantification

For densitometry measurements, it is particularly important when chemiluminescence is used as the detecting substrate that samples are in the dynamic range, requiring the acquisition of numerous exposures. An important component of quantification is verification that equal protein amounts were loaded in each lane or that differences are accounted for in the calculations. Most modern fluorescence and chemiluminescence detection software is able to show when detection is saturated, a feature not possible with X-ray film. Housekeeping proteins are often used to normalize the abundance of the protein of interest to the amount of total protein run in a lane (i.e., to the housekeeping protein). There is one note to highlight: if using a housekeeping protein to normalize across lanes, make sure that the protein is really not changing under your different conditions. For example, GAPDH protein is frequently used yet is also commonly changed under different cardiovascular conditions such as myocardial infarction (36). The issue of overloading is very common and needs attention when evaluating housekeeping proteins. Overloading is particularly seen when blots are stripped and reused for housekeeping protein analysis, as more total protein is frequently required for the protein of interest versus the more abundant housekeeping protein (40). When analyzing the same sample set for multiple primary antibodies, we recommend not stripping blots and using one blot per primary antibody. Having one blot per antibody will allow different total protein amounts to be loaded on individual gels if needed, thus avoiding overloading for more abundant proteins. Assessment of loading using a total protein stain such as Coomassie blue (for gels) or Ponceau S (for membranes) provides a better means than housekeeping protein analysis and allows normalization of each individual lane to the total protein in the lane (43). This step occurs before incubation in the blocking buffer and primary antibody. For total protein assessment, densitometry is required to verify that equal loading of protein was performed.

While densitometry values are typically graphed, it is important to show >1 representative lane for each group for both the protein of interest and the total membrane stain, to demonstrate the variability in the range of abundance that occurs in most studies, as discussed in more detail below in *Immunoblot Presentation*. Frequently, it is the relative abundance of a protein that is calculated from an immunoblot (e.g., comparing the relative abundance of a protein of interest in a control group vs. an experimental group). Values for controls are commonly presented as 100%, allowing the experimental group to be clearly presented as a percent increase or decrease compared with the control. While background correction is not recommended as an attempt to clean up very dirty membranes, background correction is useful to prevent incorrect quantification. The main criterion to use is that the densitometry values faithfully reflect the intensity of the bands on the membrane.

### Immunoblot Presentation

An area of importance is the presentation of immunoblot results. For the results section, molecular weight details of where the protein is observed in the blot should be provided. Many immunoblot figures lack molecular weight markers, which are needed to document the size where the band is observed. It is not unusual for additional bands to show up on the blot; these should be recorded either in the results text or by showing the entire blot and indicating the molecular weight of the protein of interest. This is essential to avoid confusion as to which band was quantified.

Often only one sample from each experimental group is included as a representative in the immunoblot figure, while the experimental groups usually contain many more samples (e.g., *n* = 3–8). Few details are given as to how these additional samples were compared on immunoblots and how normalization was calculated. We stress that quantification between blots is not standard practice and can raise questions from reviewers and readers unless rigorous normalization of repeat samples across blots is clearly presented. When showing the graph of the densitometry quantification, the representative sample immunoblot should ideally reflect the mean and range of the groups graphed, which would provide information on the biological variability within samples of each group (e.g., using box plots or showing individual values). When possible, we strongly recommend showing >1 sample/group, as this also provides information on response variability.

### Immunoblot Interpretation

There are a few points to remember when interpreting immunoblot results. First, the band appearing at the expected molecular weight is consistent with, but is not evidence of, specificity. Final confirmation can be obtained by using null tissue or cells or by absorbing the protein from the sample and showing that the band is no longer seen. Second, antibodies recognize epitopes, and epitope availability can be affected by conditions. Third, reduced densitometry or nondetection may not actually mean that the protein is lower or absent, but rather that the antibody is no longer recognizing the epitope. The protein could actually be present in the same amount, and the epitope is no longer present because of posttranslational modification. All of these considerations should be taken into account.

## IMMUNOHISTOCHEMISTRY, IMMUNOFLUORESCENCE, AND IMMUNOCYTOCHEMISTRY

Immunohistochemistry refers to the detection of a specific protein antigen in a histological tissue sample. Immunofluorescence is a subtype of immunohistochemistry, where the label used is fluorescent. Immunocytochemistry refers to the detection of a specific protein antigen in a histological cell sample. Indirect immunohistochemistry using an unlabeled primary antibody and a species-specific labeled secondary antibody is common. Labeling methods include three types: fluorescent, enzyme, or particulate. Of these, fluorescent and enzyme labels are the most frequently used. Histological enzymes convert a water-soluble uncolored substrate to a water-insoluble color product. Particulate labels include colloidal gold and are primarily used for electron microscopy. Further amplification of the signal can be achieved using an avidin-biotin complex (the ABC approach) after the secondary antibody to add the horseradish peroxidase enzyme to achieve high sensitivity through a method that is easy to perform (6). It is important that similar exposure times be used across samples, to ensure accurate comparisons. Examples of using the ABC approach are common in *American Journal of Physiology-Heart and Circulatory Physiology* publications (23, 31, 38, 53). There are a number of articles that have reviewed immunohistochemistry approaches (55, 57).

### Primary and Secondary Antibody Controls

While primary antibody controls are in common to both immunoblot analysis and immunohistochemistry, immunohistochemistry has additional secondary antibody and labeling controls that need to be performed when testing out a new antibody or method (for background nonspecific binding issues). Negative controls include incubating samples with no primary antibody or with an equal amount of normal serum from the same species from which the antibody was generated. The caveats to these controls include nonspecific binding of the secondary antibody and unique binding of the secondary antibody to Fc receptors, both of which can occur at injury sites and areas of inflammation when there are large numbers of leukocytes, which make this control particularly difficult to interpret for cardiovascular studies (6, 18). Likewise, labeling controls identify the contribution of endogenous fluorescence or enzymatic activity, both of which are problematic in cardiovascular tissues. For these reasons, secondary antibody and labeling controls are used most often as a subtractive appraisal rather than as an all-or-nothing analysis.

The College of American Pathologists recognizes that it is impractical to have separate positive control samples for every combination of fix, processing, and specimen type (7). If there is insufficient material to allow a full range of controls to be performed, it is recommended that antibody concentration and conditions to reduce background staining be worked out on surrogate samples for which there is plenty of material. In surgical pathology, immunohistochemistry results are considered valid only if proper positive and negative controls are used and evaluated in each procedure. While this is more easily done for immunohistochemistry using formalin-fixed tissue embedded in paraffin before sectioning and staining, it is more difficult for immunocytochemistry, where samples are frequently derived from different sources and processing can vary. At the same time, it is more important to provide rigorous documentation of the procedure and what controls were used.

### Analysis

An important consideration to make is that controls only assess whether nonspecific binding is occurring; controls will not eliminate this possibility from occurring. For example, absorption controls will evaluate whether the primary antibody is binding antigen and do not exclude the possibility that the primary antibody is binding to anything else. For immunohistochemistry, the absence of an antigen from a tissue (such as a negative source tissue) will often lead to higher nonspecific staining seen in that sample than in a tissue that has plenty of antigen present. This needs to be taken into consideration when quantification is made, to make sure that nonspecific binding labeling is not included as positive staining (49).

## RECOMMENDATIONS AND CONCLUSIONS

In a perfect world with unlimited time and resources, every control can be performed and every condition matched perfectly. In reality, not every condition for complete validation will be met. Antibodies are frequently selective only under certain conditions, very frequently lack specificity to not target any other antigen, or cannot be validated using every means possible. To increase the chance that an antibody use is reproducible, all stakeholders in the life science community need to participate. This includes societies (such as the American Physiological Society), suppliers, journal publishers, reviewers, funding agencies, institutions, and investigators.

From the supplier viewpoint, current efforts are being made to provide consensus across the field. The International Working Group for Antibody Validation was established to develop a proposal for the validation of antibodies, including an antibody scoring system (56). They proposed five conceptual pillars for antibody validation in an application-specific manner. These pillars include using the following approaches: *1*) genetic negative control using genome editing or RNA interference; *2*) orthogonal evaluation using an antibody-independent method such as mass spectrometry to sequence the protein of interest; *3*) independent second primary antibody that has nonoverlapping epitope with the first antibody; *4*) epitope-tagged protein pulldown, to show that protein levels correlate with immunoblot analysis densitometry; and *5*) immunocapture followed by mass spectrometry. Ideally, the above steps would be performed by the supplier of the antibody to confirm validity of its antibody. Some of the groups shown in Table 1 are also excellent resources to share information on the known quality of antibodies.

From the journal publisher, journal, and reviewer viewpoint, efforts should be made to ensure that the current instructions to authors are met. Developing a checklist will help reviewers to spot omissions more easily, and review by journal staff should be made at least for accepted manuscripts. Because there is a large disconnect between what is written in the journal instructions and what we observed in the actual publications, review should occur at the level of the journal, and this could occur at the prepublication proof stage. For all *American Journal of Physiology* journals, all figures are checked for potential image issues once a manuscript is accepted, and any issues identified are corrected before publication (59). From the viewpoint of funding agencies, there are ongoing discussions and efforts to increase rigor and reproducibility, and a key part of this is authentication of biological reagents (25). To date, institutions for the most part have not weighed in on these issues.

From the investigator viewpoint, the majority of the burden of proof lies with the efforts of the researcher in experimental design, implementation, and analysis. Table 3 shows a checklist of information that should be provided in the description of methods and results. In particular, details on how the methods were performed and how results were acquired and normalized are crucial. If >3 antibodies are used, a table listing each with their conditions of use will be helpful to reviewers and readers.

**Table 3.** Guidelines: minimum details to be provided in description of methods and results

*Immunoblot analysis*
Methods
• Tissue extraction: buffer reagent and protein quantification details • Concentration of protein loaded for each protein of interest • Gel: percentage SDS-PAGE and membrane type (nitrocellulose or polyvinylidene difluoride) • Primary and secondary antibodies: name, source, lot number, dilution, incubation time, and temperature • Label: type, source, incubation time, and exposure time • Documentation or reference confirming antibody-antigen binding (positive and negative controls used): type, tissue source, and processing method
Results
• Molecular weight information • Number of bands recognized • Blot should include at least three biological replicates for each group • Positive control for the antibody (e.g., protein extract of high-abundance tissue)
*Immunohistochemistry*
Methods
• Tissue fixation and processing details • Primary and secondary antibodies: name, source, lot number, dilution, incubation time, and temperature • Label: type, source, and incubation time • Documentation or reference confirming antibody-antigen binding (positive and negative controls used): type, tissue source, and processing method
Results
• Representative images, including no primary and no secondary antibody negative controls • Quantification, blinded

Modified from Gilda et al. (14) and Hewitt et al. (18).

In conclusion, this guidelines article serves to provide direction as to how to best report antibody use for immunoblot analysis and immunohistochemistry to maximize reproducibility. We also provide guidance on how to validate your antibodies to maximize the rigor of your experiment. While these recommendations are not required by the *American Journal of Physiology-Heart and Circulatory Physiology* at this time, our suggestions can help authors avoid potential validation issues with their research.

## GRANTS

We acknowledge support from the following funding agencies: National Institutes of Health Grants HL-131834, HL-075360, HL-129823, HL-051971, GM-104357, GM-115428, and GM-114833, Biomedical Laboratory Research and Development Service of the Veterans Affairs Office of Research and Development Grant 5I01BX000505, and the Sarver Heart Center, University of Arizona.

## DISCLAIMERS

The content is solely the responsibility of the authors and does not necessarily represent the official views of any of the funding agencies listed.

## DISCLOSURES

No conflicts of interest, financial or otherwise, are declared by the authors.

## AUTHOR CONTRIBUTIONS

H.L.B. and M.L.L. conceived and designed research; H.L.B. and M.L.L. performed experiments; H.L.B. and M.L.L. analyzed data; H.L.B. and M.L.L. interpreted results of experiments; H.L.B. and M.L.L. prepared figures; H.L.B. and M.L.L. drafted manuscript; H.L.B. and M.L.L. edited and revised manuscript; H.L.B. and M.L.L. approved final version of manuscript.
